# Supplementary material for: Maternal body condition and season influence RNA deposition in the oocytes of alfalfa leafcutting bees (Megachile rotundata)
Source: Front Genet. 2023 Jan 4;13:1064332. doi: 10.3389/fgene.2022.1064332 (PMC9845908; doi:10.3389/fgene.2022.1064332)
Supplement: Supplementary file 8 [file DataSheet10.pdf]

Supplementary Material

1 SUPPLEMENTARY DATA

Nine supplemental data files were included at submission and will be uploaded to FigShare for permanent storage after acceptance.

2 SUPPLEMENTARY TABLES AND FIGURES

2.1 Tables

Table S1. Oocyte tissue: key for comparing renamed, manuscript-specific and WGCNA-generated module naming patterns. WGCNA assignments correspond to module ids used in the manuscript. Egg modules were matched to oocyte modules.

Manuscript Name	Matched Name	Assigned Name
oocyteMod1	-	grey
oocyteMod2	-	saddlebrown
oocyteMod3	-	floralwhite
oocyteMod4	-	midnightblue
oocyteMod5	-	black
oocyteMod6	-	mediumpurple3
oocyteMod7	-	orangered4
oocyteMod8	-	orange
oocyteMod9	-	lightyellow
oocyteMod10	-	ivory
oocyteMod11	-	white
oocyteMod12	-	bisque4
oocyteMod13	-	skyblue3
oocyteMod14	-	darkolivegreen
oocyteMod15	-	brown4
oocyteMod16	-	grey60
oocyteMod17	-	royalblue
oocyteMod18	-	yellow
oocyteMod19	-	darkred
oocyteMod20	-	darkturquoise
oocyteMod21	-	darkgrey
oocyteMod22	-	blue
oocyteMod23	-	lightsteelblue1
oocyteMod24	-	darkorange2
oocyteMod25	-	paleturquoise
oocyteMod26	-	cyan
oocyteMod27	-	darkmagenta
oocyteMod28	-	lightcyan1
oocyteMod29	-	violet
oocyteMod30	-	sienna3

Table S2. Egg tissue: key for comparing renamed, manuscript-specific and WGCNA-generated module naming patterns. Egg module ids were matched and relabeled to the oocyte module labels, as indicated by the *matched name* column.

Manuscript Name	Matched Name	Assigned Name
eggMod1	bisque4	lightsteelblue1
eggMod2	purple	mediumpurple3
eggMod3	white	steelblue
eggMod4	lightsteelblue1	blue
eggMod5	blue	yellowgreen
eggMod6	brown4	bisque4
eggMod7	lightyellow	brown
eggMod8	darkred	lightgreen
eggMod9	pink	darkslateblue
eggMod10	sienna3	sienna3
eggMod11	red	darkorange2
eggMod12	floralwhite	darkorange
eggMod13	darkgrey	lightcyan1
eggMod14	grey	grey
eggMod15	orange	darkgey
eggMod16	saddlebrown	thistle1
eggMod17	midnightblue	darkmagenta
eggMod18	paleturquoise	plum2
eggMod19	magenta	darkturquoise
eggMod20	cyan	greenyellow
eggMod21	black	cyan
eggMod22	orangered4	floralwhite
eggMod23	royalblue	plum1
eggMod24	darkturquoise	green
eggMod25	grey60	midnightblue
eggMod26	turquoise	brown4
eggMod27	skyblue3	thistle2

Table S3. Enrichment results ($p < 0.05$) for genes that were significantly differentially expressed with maternal body condition in oocyte tissue. For full results, see Supplemental File 2.

GO.ID	Terms	Annotated	Significant	Expected	*p*-value
Biological Processes:					
GO:0006506	GPI anchor biosynthetic process	21	0	0.06	0.011
GO:0006388	tRNA splicing, via endonucleolytic cleavage and ligation	3	0	0.01	0.011
GO:0006488	dolichol-linked oligosaccharide biosynthetic process	8	0	0.02	0.011
GO:0006457	protein folding	30	0	0.09	0.014
GO:0006412	translation	194	0	0.56	0.015
GO:0071526	semaphorin-plexin signaling pathway	3	0	0.01	0.022
GO:0003341	cilium movement	6	0	0.02	0.026
GO:0006122	mitochondrial electron transport, ubiquinol to cytochrome c	5	0	0.01	0.032
GO:0030488	tRNA methylation	3	0	0.01	0.032
GO:0090263	positive regulation of canonical Wnt signaling pathway	2	0	0.01	0.034
GO:0032049	cardiolipin biosynthetic process	2	0	0.01	0.035
GO:0034220	ion transmembrane transport	70	0	0.2	0.036
GO:0000079	regulation of cyclin-dependent protein serine/threonine kinase activity	3	0	0.01	0.039
GO:0006508	proteolysis	210	1	0.61	0.042
Molecular Function:					
GO:0036402	proteasome-activating activity	5	0	0.02	0.00014
GO:0030234	enzyme regulator activity	141	1	0.51	0.0002
GO:0016887	ATP hydrolysis activity	60	0	0.21	0.01022
GO:0140662	ATP-dependent protein folding chaperone	19	0	0.07	0.01109
GO:0004252	serine-type endopeptidase activity	41	0	0.15	0.01246
GO:0015267	channel activity	78	0	0.28	0.01818
GO:0003735	structural constituent of ribosome	120	0	0.43	0.01852
GO:0017154	semaphorin receptor activity	3	0	0.01	0.02227
GO:0016538	cyclin-dependent protein serine/threonine kinase regulator activity	12	0	0.04	0.02559
GO:0019843	rRNA binding	14	0	0.05	0.0301
GO:0051879	Hsp90 protein binding	5	0	0.02	0.03051
GO:0004867	serine-type endopeptidase inhibitor activity	11	0	0.04	0.03167
GO:0060072	large conductance calcium-activated potassium channel activity	3	0	0.01	0.03183
GO:0004376	glycolipid mannosyltransferase activity	5	0	0.02	0.03477
GO:0016772	transferase activity, transferring phosphorus-containing groups	322	1	1.15	0.03799
GO:0016798	hydrolase activity, acting on glycosyl bonds	40	0	0.14	0.03931
GO:0005230	extracellular ligand-gated ion channel activity	24	0	0.09	0.0427
GO:0004298	threonine-type endopeptidase activity	2	0	0.01	0.04404
GO:0000213	tRNA-intron endonuclease activity	2	0	0.01	0.04765
GO:0051082	unfolded protein binding	17	0	0.06	0.04893
GO:0016757	glycosyltransferase activity	84	1	0.3	0.04894
Cellular Components:					
GO:0000439	transcription factor TFIIH core complex	4	0	0.01	0.0059
GO:0005576	extracellular region	46	2	0.16	0.0203
GO:0019773	proteasome core complex, alpha-subunit complex	7	1	0.02	0.0208
GO:0005839	proteasome core complex	14	1	0.05	0.0239
GO:0062023	collagen-containing extracellular matrix	2	0	0.01	0.0273
GO:0046658	anchored component of plasma membrane	2	0	0.01	0.0273
GO:0005887	integral component of plasma membrane	9	0	0.03	0.0321
GO:0005750	mitochondrial respiratory chain complex III	5	0	0.02	0.0334
GO:0005840	ribosome	121	0	0.42	0.0486

Table S4. Enrichment results ($p < 0.05$) for genes that were significantly differentially expressed with time of season in oocyte tissue. For full results, see Supplemental File 2.

GO.ID	Terms	Annotated	Significant	Expected	*p*-value
Biological Processes:					
GO:1902600	proton transmembrane transport	26	6	3.03	0.000028
GO:0006468	protein phosphorylation	199	27	23.21	0.00087
GO:0006355	regulation of transcription, DNA-templated	226	36	26.36	0.00136
GO:0051056	regulation of small GTPase mediated signal transduction	14	1	1.63	0.00607
GO:0006470	protein dephosphorylation	33	8	3.85	0.00611
GO:0016485	protein processing	13	3	1.52	0.00907
GO:0046488	phosphatidylinositol metabolic process	34	4	3.97	0.02429
GO:0017121	plasma membrane phospholipid scrambling	2	2	0.23	0.02636
GO:0006821	chloride transport	4	1	0.47	0.02705
GO:0070588	calcium ion transmembrane transport	9	3	1.05	0.03141
GO:0006897	endocytosis	12	1	1.4	0.03442
GO:0010506	regulation of autophagy	2	2	0.23	0.03738
GO:0006874	cellular calcium ion homeostasis	7	3	0.82	0.03857
GO:0042176	regulation of protein catabolic process	5	0	0.58	0.04374
GO:0032469	endoplasmic reticulum calcium ion homeostasis	2	1	0.23	0.04806
Molecular Function:					
GO:0004725	protein tyrosine phosphatase activity	17	6	2.12	0.00028
GO:0046961	proton-transporting ATPase activity, rotational mechanism	11	6	1.37	0.00122
GO:0005198	structural molecule activity	161	10	20.12	0.00639
GO:0004672	protein kinase activity	199	26	24.86	0.0122
GO:0008483	transaminase activity	12	1	1.5	0.01805
GO:0046912	acyltransferase, acyl groups converted into alkyl on transfer	3	2	0.37	0.02237
GO:0004190	aspartic-type endopeptidase activity	4	2	0.5	0.02319
GO:0030276	clathrin binding	5	1	0.62	0.0235
GO:0017128	phospholipid scramblase activity	2	2	0.25	0.02746
GO:0016307	phosphatidylinositol phosphate kinase activity	3	2	0.37	0.02791
GO:0005247	voltage-gated chloride channel activity	3	1	0.37	0.03203
GO:0016810	hydrolase activity, acting on carbon-nitrogen (but not peptide) bonds	22	1	2.75	0.03279
GO:0004523	RNA-DNA hybrid ribonuclease activity	3	1	0.37	0.0337
GO:0009922	fatty acid elongase activity	9	2	1.12	0.03582
GO:0004867	serine-type endopeptidase inhibitor activity	11	0	1.37	0.0371
Cellular Components:					
GO:0005856	cytoskeleton	89	8	8.73	0.008
GO:0000139	Golgi membrane	9	1	0.88	0.018
GO:0016020	membrane	744	85	73.02	0.02
GO:0005765	lysosomal membrane	5	1	0.49	0.026
GO:0031932	TORC2 complex	3	1	0.29	0.035
GO:0000502	proteasome complex	21	0	2.06	0.038
GO:0000159	protein phosphatase type 2A complex	3	2	0.29	0.039
GO:0008180	COP9 signalosome	7	1	0.69	0.043
GO:0033177	proton-transporting two-sector ATPase complex, proton-transporting domain	13	3	1.28	0.043
GO:0005783	endoplasmic reticulum	61	9	5.99	0.045
GO:0005802	trans-Golgi network	4	1	0.39	0.048

Table S5. Enrichment results ($p < 0.05$) for genes from oocyte samples that were assigned to *OocyteMod29* via WGCNA analysis. *OocyteMod29* was significantly associated with time of season in oocyte tissue. For full results, see Supplemental File 8.

GO.ID	Terms	Annotated	Significant	Expected	*p*-value
Biological Processes:					
GO:1902600	proton transmembrane transport	29	7	0.25	2.20×10^{-9}
GO:0045596	negative regulation of cell differentiation	1	1	0.01	0.0085
GO:0001709	cell fate determination	1	1	0.01	0.0085
GO:0001678	cellular glucose homeostasis	2	1	0.02	0.017
GO:1904668	positive regulation of ubiquitin protein ligase activity	3	1	0.03	0.0254
GO:0035434	copper ion transmembrane transport	3	1	0.03	0.0254
GO:0006879	cellular iron ion homeostasis	4	1	0.03	0.0337
GO:0006826	iron ion transport	4	1	0.03	0.0337
GO:0000122	negative regulation of transcription by RNA polymerase II	5	1	0.04	0.042
Molecular Function:					
GO:0046961	proton-transporting ATPase activity, rotational mechanism	11	6	0.09	1.10×10^{-9}
GO:0030215	semaphorin receptor binding	6	2	0.05	0.001
GO:0015078	proton transmembrane transporter activity	34	8	0.28	0.012
GO:0005536	glucose binding	2	1	0.02	0.017
GO:0004396	hexokinase activity	2	1	0.02	0.017
GO:0005375	copper ion transmembrane transporter activity	3	1	0.02	0.025
GO:0097027	ubiquitin-protein transferase activator activity	3	1	0.02	0.025
GO:0010997	anaphase-promoting complex binding	3	1	0.02	0.025
GO:0004861	cyclin-dependent protein serine/threonine kinase inhibitor activity	4	1	0.03	0.033
GO:0008199	ferric iron binding	5	1	0.04	0.041
GO:0016298	lipase activity	41	2	0.34	0.045
Cellular Components:					
GO:0033177	proton-transporting two-sector ATPase complex, proton-transporting domain	13	3	0.09	0.0015
GO:0016471	vacuolar proton-transporting V-type ATPase complex	2	2	0.01	0.0068
GO:0000221	vacuolar proton-transporting V-type ATPase, V1 domain	1	1	0.01	0.0073
GO:0033178	proton-transporting two-sector ATPase complex, catalytic domain	6	2	0.04	0.0269
GO:0033179	proton-transporting V-type ATPase, V0 domain	4	1	0.03	0.0288

Table S6. Enrichment results ($p < 0.05$) for genes from oocyte samples that were assigned to *OocyteMod20* via WGCNA analysis. *OocyteMod20* was significantly associated with time of season in oocyte tissue. For full results, see Supplemental File 8.

GO.ID	Terms	Annotated	Significant	Expected	*p*-value
Biological Processes:					
GO:0016192	vesicle-mediated transport	131	24	9.96	0.0038
GO:0006429	leucyl-tRNA aminoacylation	2	2	0.15	0.0058
GO:0018216	peptidyl-arginine methylation	7	3	0.53	0.0121
GO:0006829	zinc ion transport	3	2	0.23	0.0164
GO:0009058	biosynthetic process	859	72	65.31	0.0247
GO:0006888	endoplasmic reticulum to Golgi vesicle-mediated transport	16	4	1.22	0.0287
GO:0006750	glutathione biosynthetic process	4	2	0.3	0.0312
GO:0006379	mRNA cleavage	4	2	0.3	0.0312
GO:0002098	tRNA wobble uridine modification	10	3	0.76	0.0349
GO:0006886	intracellular protein transport	83	11	6.31	0.0399
GO:0006779	porphyrin-containing compound biosynthetic process	6	3	0.46	0.0491
GO:0042176	regulation of protein catabolic process	5	2	0.38	0.0494
Molecular Function:					
GO:0004363	glutathione synthase activity	2	2	0.14	0.0052
GO:0004823	leucine-tRNA ligase activity	2	2	0.14	0.0052
GO:0016274	protein-arginine N-methyltransferase activity	7	3	0.51	0.0105
GO:0032549	ribonucleoside binding	3	2	0.22	0.0148
GO:0003735	structural constituent of ribosome	120	15	8.66	0.0248
GO:0015299	solute:proton antiporter activity	4	2	0.29	0.0283
GO:0017025	TBP-class protein binding	4	2	0.29	0.0283
GO:0070122	isopeptidase activity	10	3	0.72	0.0305
GO:0008083	growth factor activity	18	4	1.3	0.0363
GO:0005544	calcium-dependent phospholipid binding	5	2	0.36	0.0449
GO:0003887	DNA-directed DNA polymerase activity	5	2	0.36	0.0449
Cellular Components:					
GO:0000502	proteasome complex	21	6	1.52	0.014
GO:0072546	EMC complex	3	2	0.22	0.015
GO:0030008	TRAPP complex	5	3	0.36	0.028
GO:0005869	dynactin complex	4	2	0.29	0.028
GO:0005763	mitochondrial small ribosomal subunit	4	2	0.29	0.028
GO:0005765	lysosomal membrane	5	2	0.36	0.045

Table S7. Enrichment results ($p < 0.05$) for genes from oocyte samples that were assigned to *OocyteMod16* via WGCNA analysis. *OocyteMod16* was significantly associated with time of season in oocyte tissue. For full results, see Supplemental File 8.

GO.ID	Terms	Annotated	Significant	Expected	*p*-value
Biological Processes:					
GO:0006813	potassium ion transport	36	8	1.8	0.0039
GO:0045893	positive regulation of transcription, DNA-templated	16	4	0.8	0.0069
GO:0007160	cell-matrix adhesion	3	2	0.15	0.0073
GO:0007186	G protein-coupled receptor signaling pathway	114	12	5.72	0.0189
GO:0071805	potassium ion transmembrane transport	22	4	1.1	0.022
GO:0006801	superoxide metabolic process	5	2	0.25	0.0226
GO:0019752	carboxylic acid metabolic process	113	7	5.67	0.0248
GO:0006811	ion transport	198	22	9.93	0.0322
GO:0055085	transmembrane transport	355	29	17.8	0.0483
GO:0060271	cilium assembly	29	6	1.45	0.0485
Molecular Function:					
GO:0005242	inward rectifier potassium channel activity	2	2	0.12	0.0036
GO:0005506	iron ion binding	64	10	3.83	0.0043
GO:0005515	protein binding	1507	117	90.17	0.0047
GO:0005509	calcium ion binding	120	15	7.18	0.0049
GO:0020037	heme binding	67	10	4.01	0.006
GO:0070840	dynein complex binding	3	2	0.18	0.0103
GO:0004497	monooxygenase activity	61	11	3.65	0.012
GO:0046983	protein dimerization activity	108	12	6.46	0.013
GO:0050661	NADP binding	9	3	0.54	0.0136
GO:0005267	potassium channel activity	25	7	1.5	0.0157
GO:0004842	ubiquitin-protein transferase activity	66	7	3.95	0.027
GO:0004930	G protein-coupled receptor activity	98	11	5.86	0.0312
GO:0005201	extracellular matrix structural constituent	5	2	0.3	0.0316
GO:0016705	oxidoreductase activity, acting on paired donors, with incorporation or reduction of molecular oxygen	72	10	4.31	0.0377
Cellular Components:					
GO:0016021	integral component of membrane	519	44	29.21	0.0021
GO:0005861	troponin complex	2	2	0.11	0.0031
GO:0005581	collagen trimer	2	2	0.11	0.0031
GO:0016020	membrane	1008	75	56.73	0.0122
GO:0034464	BBSome	6	2	0.34	0.0406
GO:0005576	extracellular region	73	8	4.11	0.0443

Table S8. Enrichment results ($p < 0.05$) for genes from oocyte samples that were assigned to *OocyteMod4* via WGCNA analysis. *OocyteMod4* was significantly associated with time of season in oocyte tissue. For full results, see Supplemental File 8.

GO.ID	Terms	Annotated	Significant	Expected	*p*-value
Biological Processes:					
GO:0071929	alpha-tubulin acetylation	1	1	0.01	0.014
GO:0043063	intercellular bridge organization	1	1	0.01	0.014
GO:0002218	activation of innate immune response	1	1	0.01	0.014
GO:0007300	ovarian nurse cell to oocyte transport	1	1	0.01	0.014
GO:0032481	positive regulation of type I interferon production	1	1	0.01	0.014
GO:0007140	male meiotic nuclear division	1	1	0.01	0.014
GO:0006814	sodium ion transport	14	2	0.19	0.015
GO:0007608	sensory perception of smell	43	3	0.59	0.021
GO:0008608	attachment of spindle microtubules to kinetochore	2	1	0.03	0.027
GO:0035434	copper ion transmembrane transport	3	1	0.04	0.041
GO:1905515	non-motile cilium assembly	3	1	0.04	0.041
GO:0006098	pentose-phosphate shunt	3	1	0.04	0.041
Molecular Function:					
GO:0046961	proton-transporting ATPase activity, rotational mechanism	11	2	0.16	0.011
GO:0009922	fatty acid elongase activity	12	2	0.18	0.013
GO:0004801	transaldolase activity	1	1	0.01	0.015
GO:0008113	peptide-methionine (S)-S-oxide reductase activity	1	1	0.01	0.015
GO:0019799	tubulin N-acetyltransferase activity	1	1	0.01	0.015
GO:0080019	fatty-acyl-CoA reductase (alcohol-forming) activity	13	2	0.19	0.015
GO:0051015	actin filament binding	16	2	0.24	0.023
GO:0004984	olfactory receptor activity	43	3	0.64	0.025
GO:0140575	transmembrane monodehydroascorbate reductase activity	2	1	0.03	0.029
GO:0004471	malate dehydrogenase (decarboxylating) (NAD+) activity	2	1	0.03	0.029
GO:0004731	purine-nucleoside phosphorylase activity	2	1	0.03	0.029
GO:0005549	odorant binding	50	3	0.74	0.037
GO:0005375	copper ion transmembrane transporter activity	3	1	0.04	0.044
GO:0003727	single-stranded RNA binding	3	1	0.04	0.044
Cellular Components:					
GO:0033180	proton-transporting V-type ATPase, V1 domain	2	1	0.03	0.027
GO:0005890	sodium:potassium-exchanging ATPase complex	3	1	0.04	0.04

Table S9. WGCNA modules for oocyte tissue that are significantly associated with time of season, and the corresponding modules for egg tissue with a significant number of overlapping genes. The number of genes in each oocyte module and their correlation with time of season (column 2), as well as the number of genes in each egg modules and their relationship with time of season (column 4) are provided. For oocyte module-egg module pairs, the amount of genes within each oocyte modules that overlap with each egg module are reported as percentages (i.e., of overlapping genes/ number of genes in each oocyte module). p-values and representation factors (RF) are given for each module-module pair. Asterisk indicate p-values of correlation with time of season: $*p < 0.05$, $**p < 0.01$, $**p < 0.001$

	**# genes (*r* with season)**	**Egg Module**	**# genes (*r* with season)**	**% Overlap (with Egg Module)**	**Overlap *p*-value**	**RF**
OocyteMod16	588 (-0.46*)	eggMod25	230 (0.18)	4.1	0.00043	2.115
		eggMod4	4031 (-0.28)	51.9	2.08×10^{-20}	1.534
		eggMod17	330 (-0.084)	4.3	0.02191	1.536
		eggMod20	289 (0.079)	3.6	0.049	1.473
OocyteMod4	175 (-0.57**)	eggMod13	78 (0.2)	2.3	0.02765	3.492
		eggMod4	4031 (-0.28)	45.1	0.00114	1.335
		eggMod17	330 (-0.084)	5.7	0.0238	2.064
		eggMod15	192 (-0.29)	6.3	0.00012	3.902
OocyteMod29	92 (-0.51*)	eggMod4	4031 (-0.28)	65.2	6.84×10^{-10}	1.928
OocyteMod20	716 (-0.45*)	eggMod24	865 (-0.089)	25	1.45×10^{-53}	3.445
		eggMod5	645 (0.034)	12	1.10×10^{-12}	2.219
		eggMod23	287 (-0.18)	4.2	0.00216	1.74

2.2 Figures

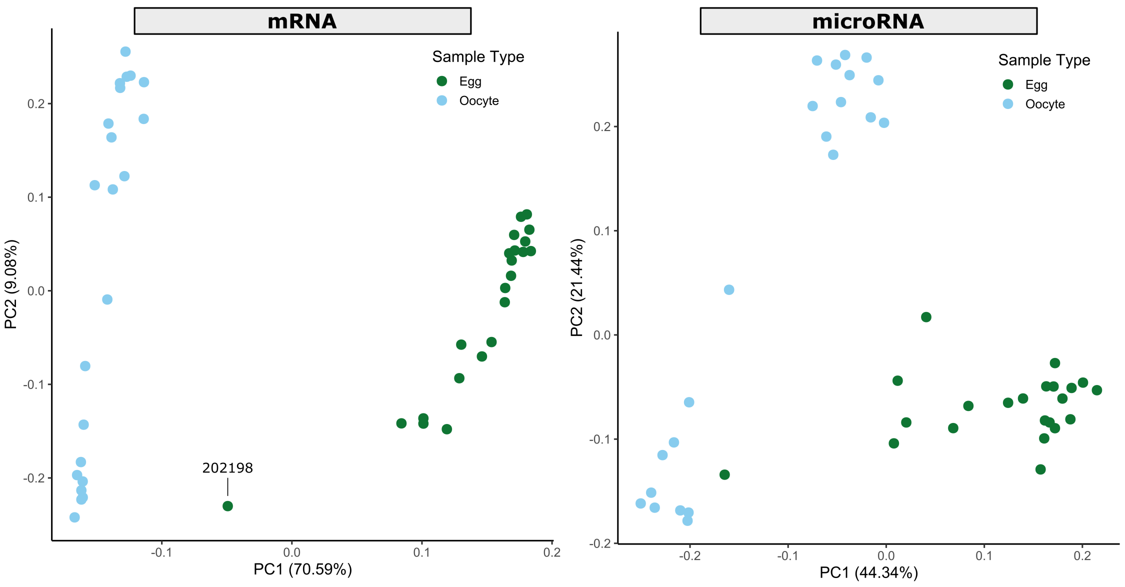

Figure S1. Principal component analysis (PCA) plots for mRNA and microRNA data showing variance between individual egg (green) and oocyte (blue) tissue samples. Egg samples were 24 h post-oviposition; oocytes were in stage 4 of maturation. Sample 202198 was identified as an outlier and removed from subsequent WGCNA analyses.

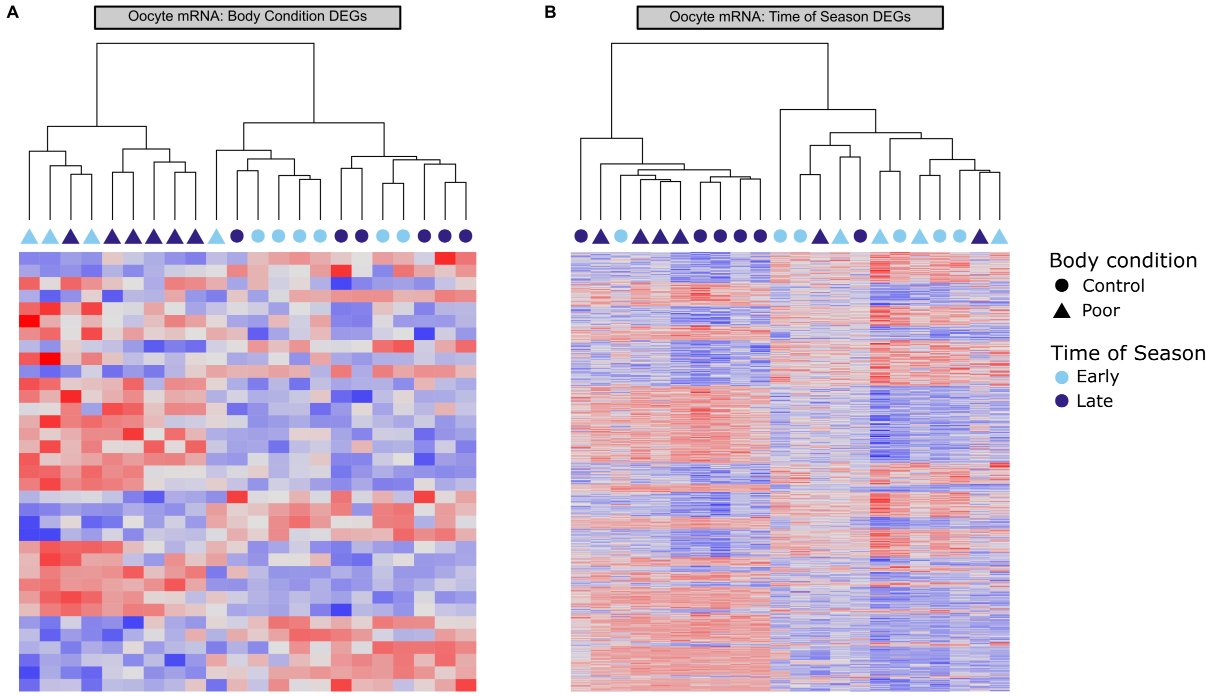

Figure S2. Significantly differentially expressed genes in oocyte tissue associated with (A) body condition and (B) photoperiod. Samples (x-axis) were either control (circle) or treatment (triangle) bees collected during the early (light blue) and late (dark blue) season. Expression key: up-regulated (red) and down-regulated (blue).

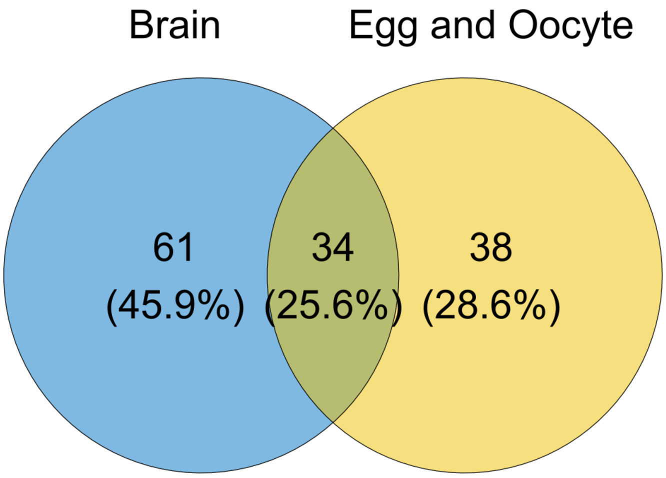

Figure S3. Venn Diagram depicting the overlap between *Megachile rotundata* brain miRNAs (Kapheim et al., 2020) and the egg/oocyte miRNAs identified in the current study.

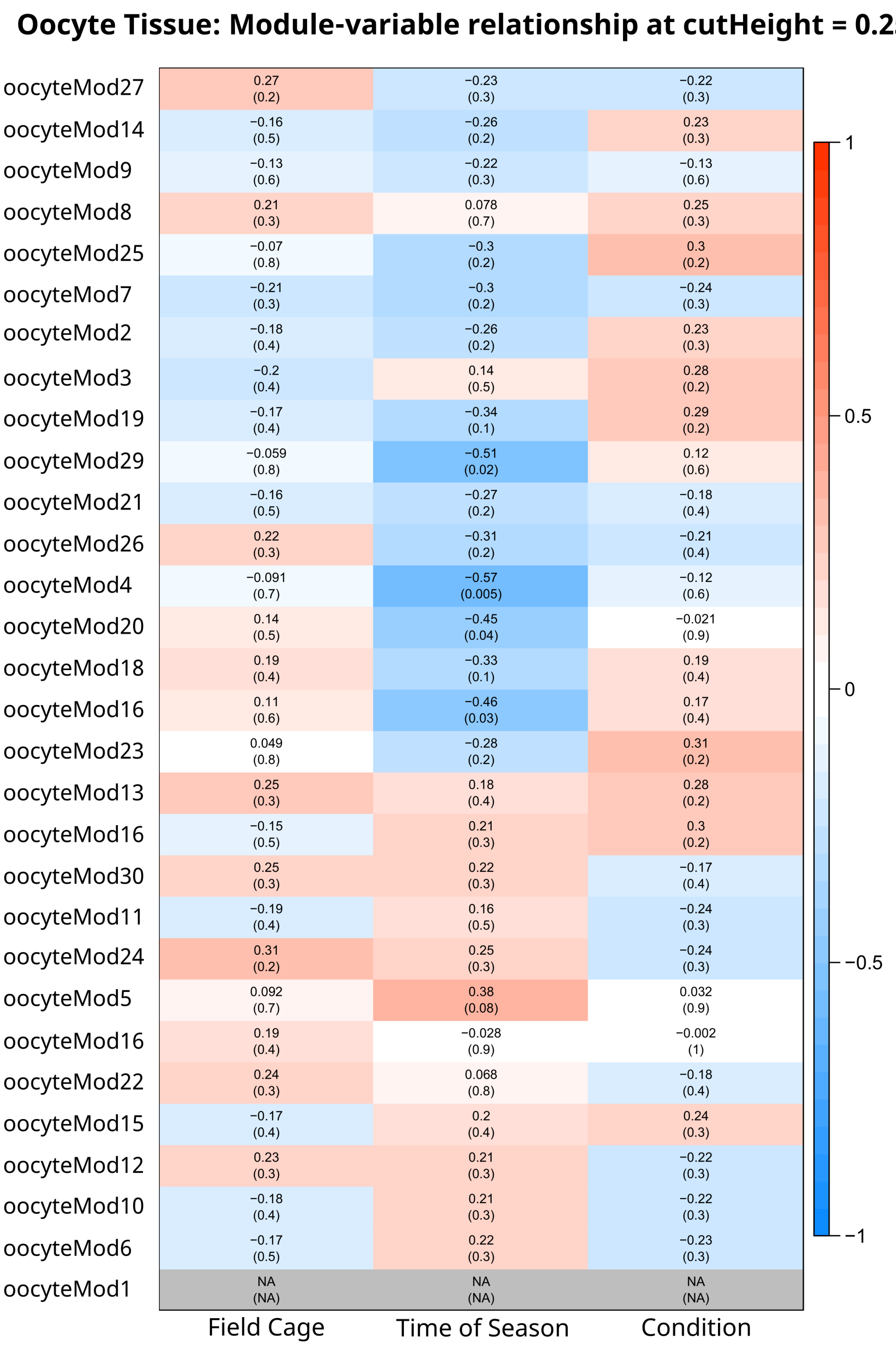

Figure S4. Correlation matrix between WGCNA gene modules (cut height = 0.25) and experimental variables for oocyte tissue. Correlation coefficients for each module-variable relationship, and their significance, are shown as r (p-value) and indicated using the color scale (positive relationship=red; negative relationship=blue; no relationship=white).

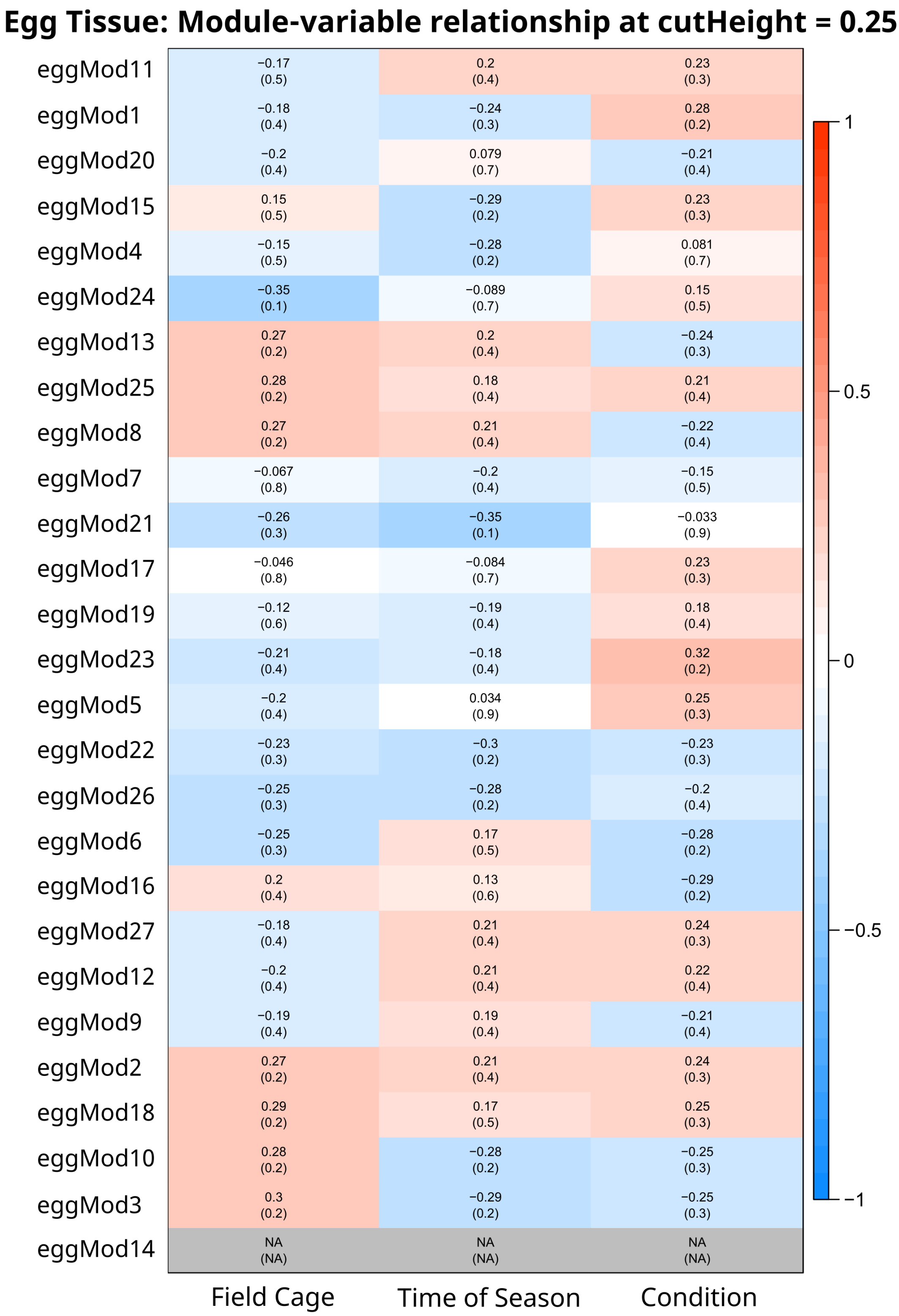

Figure S5. Correlation matrix between WGCNA gene modules (cut height = 0.25) and experimental variables for egg tissue. Correlation coefficients for each module-variable relationship, and their significance, are shown as r (p-value) and indicated using the color scale (positive relationship=red; negative relationship=blue; no relationship=white).

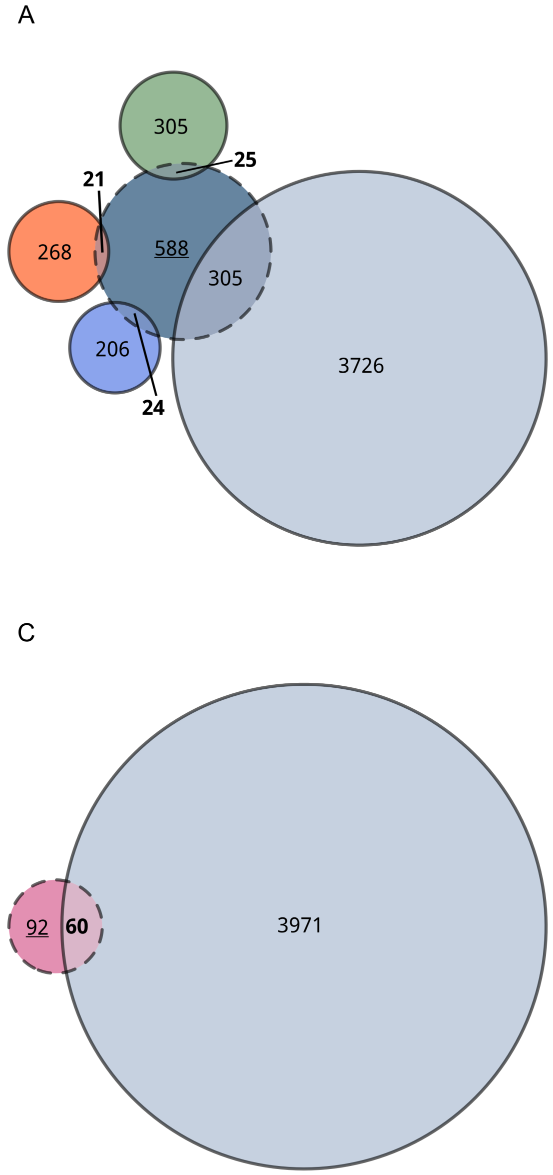

Figure S6. Oocyte modules of interest (i.e., correlated with time of season) and egg modules with which they have significant overlap. Oocyte modules, including (A) *oocyteMod16*, (B) *oocyteMod4*, (C) *oocyteMod29*, and (D) *oocyteMod20* are indicated using the dashed lines. The actual gene counts per oocyte module are underlined. Egg modules with significant overlap are indicated using solid lines and their gene counts, minus the overlap number, are present in their respective circle. The number of genes with overlap are bold and indicated at the overlapping space.

REFERENCES

Kapheim, K. M., Jones, B. M., Søvik, E., Stolle, E., Waterhouse, R. M., Bloch, G., et al. (2020). Brain micrornas among social and solitary bees. *Royal Society Open Science* 7, 200517. doi:10.1098/rsos.200517
